# Supplementary material for: The Important Role of Membrane Fluidity on the Lytic Mechanism of the α-Pore-Forming Toxin Sticholysin I
Source: Toxins (Basel). 2023 Jan 16;15(1):80. doi: 10.3390/toxins15010080 (PMC9865829; doi:10.3390/toxins15010080)
Supplement: Supplementary file 1 [file toxins-15-00080-s001.zip › toxins-2055394-supplementary.pdf]

Supplementary Materials: The Important Role of Membrane Fluidity on the Lytic Mechanism of the α-Pore-Forming Toxin Sticholysin I

Lohans Pedrera, Uris Ros, Maria Laura Fanani, María E. Lanio, Richard M. Epand, Ana J. García-Sáez and Carlos Álvarez

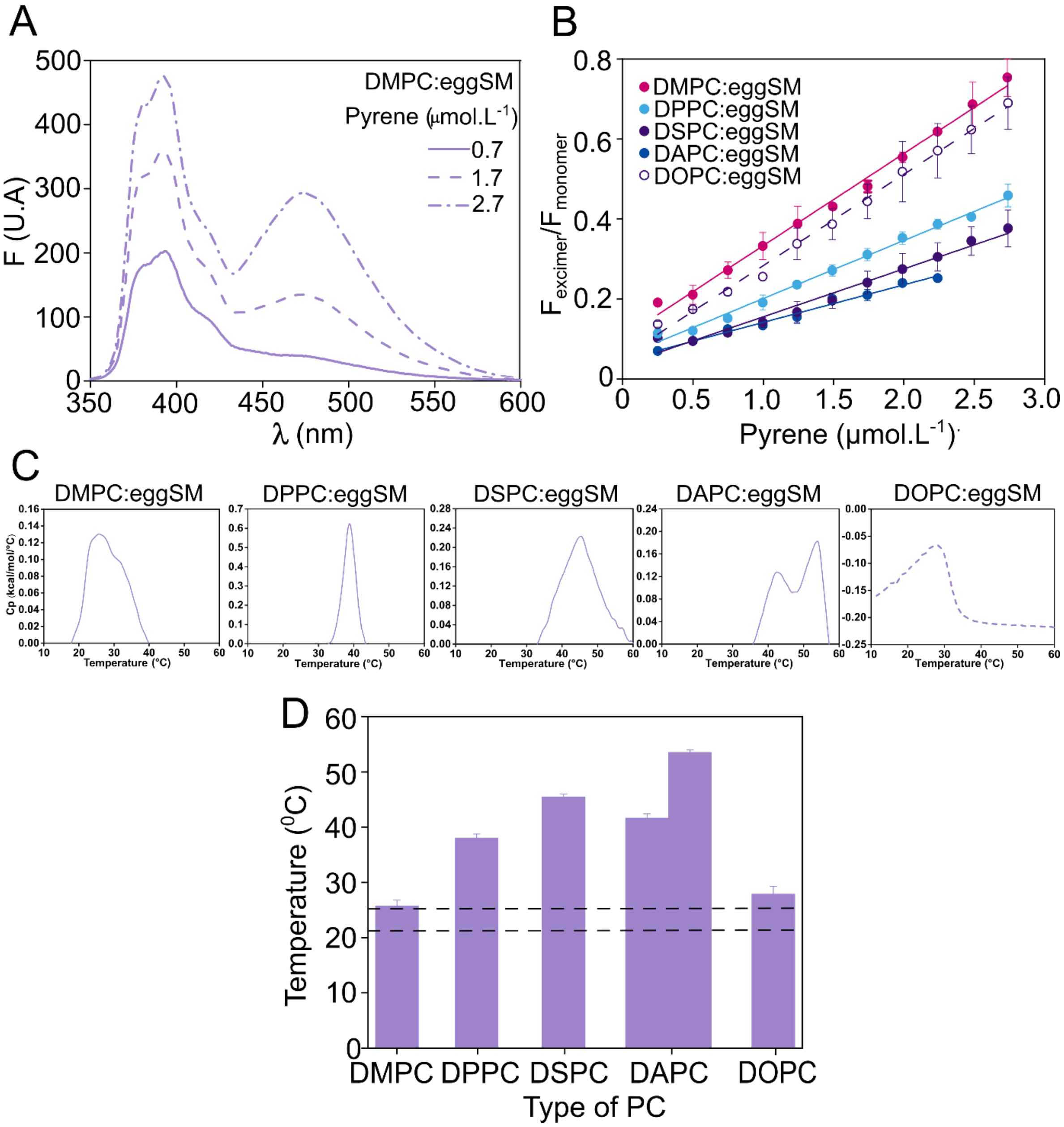

Figure S1: SUVs of PC:eggSM (50:50) containing the shorter or unsaturated PC have higher membrane fluidity.

A) Pyrene emission spectrum in SUVs composed of DMPC:eggSM (50:50). B) Effect of the type of PC on the $F_{excimer}/F_{monomer}$ ratio as a function of pyrene concentration. C) DSC heating thermograms of MLV made of PC:eggSM mixtures. Excess heat capacity (Cp) vs temperature (°C). Lipid concentration: 1 mM. Buffer solution: PIPES (20 mM PIPES, 140 mM NaCl, 1 mM EDTA, pH 7.4). Scan rate: 1 °C.min^{-1}. D) Temperature at which the

chain melting transition (Tm) of the vesicles takes place. The Tm was calculated from a heating scan obtained after reaching equilibrium upon previous heating and cooling cycles. Dotted lines indicate the range of temperature used in the functional characterization of the protein. Results are expressed as the mean and SD of three independent experiments.

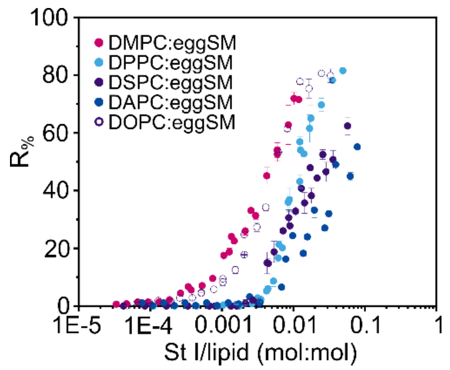

Figure S2: Effect of PC acyl chain length and unsaturation in the permeabilization activity of St I on SUVs of PC:eggSM (50:50). Dose-dependence of CF release induced by St I after 10 minutes of toxin addition. Points are the mean value, and bars indicate the standard deviation from a set of three independent experiments. When no error bar is observed the corresponding standard deviation is smaller than the size of the symbol.

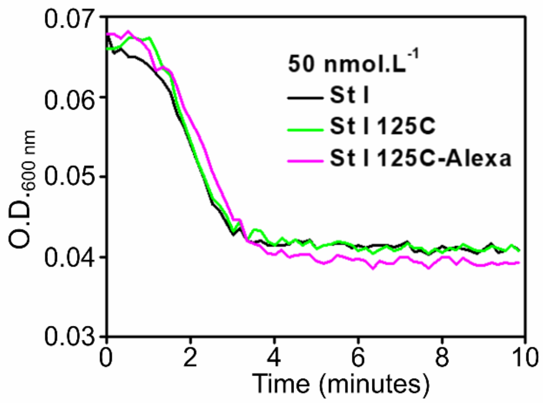

Figure S3. Hemolytic activity of native St I, the single Cys mutant St I 125 C and labeled St I 125C-Alexa. The time course of hemolysis was followed by the decrease in turbidity of a red blood cell suspension initially adjusted to an optical density of 0.07 at 600 nm.

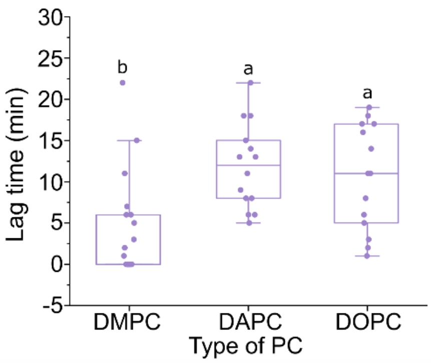

Figure S4. Effect of the PC on the lag time between the exposition of GUVs to St I and the beginning of permeabilization. This parameter was calculated from single kinetics of GUVs permeabilization as the ones showed in Figure 3B-D. Each dot represents one individual vesicle. Statistical analysis was performed with one-way ANOVA with Tukey as post-hoc test. The letters a and b indicate independent groups with significant differences among them ($p < 0.01$).

Table S1. Effect of the PC acyl chain length and unsaturation on the size of SUVs made of PC:eggSM (50:50).

**Lipid composition**	**Diameter (nm)**	**Polydispersity Index**
DPPC:eggSM	43,2 ± 0,26	0,278 ± 0,003
DSPC:eggSM	47,37 ± 0,81	0,239 ± 0,012
DAPC:eggSM	54,73 ± 0,40	0,234 ± 0,002
DOPC:eggSM	45,97 ± 0,50	0,261 ± 0,016

The size of the vesicles was determined by dynamic light scattering (Delsa Tm Nano, Beckman Coulter). The Cumulant analysis provided by the software of the equipment was used to calculate the mean diameter and the polydispersity index of the vesicles. Values are the mean and SD of at least three independent experiments.
